# The Chicken Embryo: An Old but Promising Model for In Vivo Preclinical Research

**DOI:** 10.3390/biomedicines12122835

**Published:** 2024-12-13

**Authors:** Annachiara Sarnella, Ylenia Ferrara, Cristina Terlizzi, Sandra Albanese, Serena Monti, Luca Licenziato, Marcello Mancini

**Affiliations:** Institute of Biostructures and Bioimaging, National Research Council, 80145 Naples, Italy; annachiara.sarnella@ibb.cnr.it (A.S.); ylenia.ferrara@ibb.cnr.it (Y.F.); sandra.albanese@ibb.cnr.it (S.A.); serena.monti@ibb.cnr.it (S.M.); luca.licenziato@ibb.cnr.it (L.L.); marcello.mancini@cnr.it (M.M.)

**Keywords:** chicken embryos, chorioallantoic membrane, animal model, molecular imaging, preclinical research

## Abstract

The chicken embryo has emerged as a valuable model for preclinical studies due to its unique combination of accessibility, affordability, and relevance to human biology. Its rapid development, external growth environment, and clear structural visibility offer distinct advantages over traditional mammalian models. These features facilitate the study of real-time biological processes, including tissue development, tumor growth, angiogenesis, and drug delivery, using various imaging modalities, such as optical imaging, magnetic resonance imaging, positron emission tomography, computed tomography, and ultrasound. The chicken embryo model also minimizes ethical concerns compared to mammalian models, as it allows for early-stage research without the complexity of a fully developed animal. Moreover, its ability to integrate human tumor cells into xenograft models provides a reliable platform for cancer research, enabling high-throughput screening of therapeutic interventions and tracking molecular dynamics in vivo. Advances in molecular imaging techniques further enhance the resolution and depth of data obtained from these studies, offering insights into cellular and molecular mechanisms underlying disease. Given its versatility, cost-effectiveness, and translational potential, the chicken embryo represents a promising tool for advancing preclinical research, particularly in drug development, cancer biology, and regenerative medicine.

## 1. Introduction

The chicken embryo (CE) is considered an important model that over time has contributed to the development of valuable concepts in immunology, genetics, virology, cancer, and cell biology [1]. Historically, the CE attracted the interest of the ancient Egyptians and Aristotle, who opened eggs at different stages of incubation to examine their developmental progression [2]. Over the years, a few pioneers (Rawles, Fell, Wetzel, etc.), through studies of embryonic development, perfected embryo culture and microsurgery methods, making the CE model approach accessible [1]. The first comprehensive atlas of chick morphology was published in 1889 [3], and in 1951, Hamburger and Hamilton studied the embryonic development of the CE, describing 46 distinct morphological phases, divided according to the number of stages rather than the incubation time [4].

This model is relatively simple, rapid, and low-cost, and it offers unique opportunities to study embryonic development, genetic regulation, disease mechanisms, and the effects of various substances in a controlled environment.

The model can be manipulated and cultured in most laboratories as, in the early stages, it can be considered a 3D tissue culture model that does not require ethical approval. Because of its short development period, results can be quickly obtained, significantly reducing the time and cost of research. The easy handling of the CE facilitates direct visualization of stability, biocompatibility, circulation, release, degradation, and therapeutic efficacy, key factors that predict in vivo performance in rodents and higher-order mammals. In addition, CEs are accessible for manipulation and injections under a stereomicroscope, followed by in vivo imaging in ovo and non-invasive monitoring during later development [5]. It is an established model for tissue/cell transplantation, and due to the lack of an immune system in the early stages of development, the CE is increasingly recognized as a model of choice for mammalian biology, with new applications for stem cell and cancer research. Due to their operability and intrinsic immunotolerance, CEs have been used in a series of studies to evaluate the proliferation, differentiation, and migration capacity of rodent and human stem cells [6]. This model has been used to monitor the growth and metastatic properties of various cancer cell lines grafted on the chorioallantoic membrane (CAM), which is an excellent method to study tumor metastasis [7,8]. In addition, transplantation of cancer cell lines into the CE, which can be coupled with new non-invasive live monitoring methods, represents a powerful new tool to identify signaling pathways controlling tumorigenesis and to validate new targets for therapy [5].

Accordingly, this model represents a promising alternative to traditional mouse models, in line with the ethical principles of the 3Rs (Replacement, Reduction, and Refinement), as extensively reported in ancient studies. However, its full potential in applications using molecular imaging methods remains underexplored and requires further optimization.

In the light of this, the aim of our review was not only to highlight the fundamental and established features of the CE model but also to analyze its current applications. Indeed, molecular imaging has the potential to transform research in various areas, such as drug development, biomarker discovery, and regenerative medicine, advancing more ethical and innovative practices in biomedical science. Here, by integrating the CE model with molecular imaging methods, we aim to illustrate how these technologies can open new paths for the advancement of preclinical research.

## 2. The Chicken Genome

The chicken genome is a landmark in genomics and evolutionary biology, representing the first avian genome to be sequenced and serving as a model system for research in agriculture, developmental biology, and comparative genomics. It has become a keystone for understanding vertebrate evolution, bridging the gap between mammalian and other non-mammalian genomes. The study of the chicken genome dates back to 1936, when the first genetic linkage map was constructed, making the chicken one of the earliest species to have its genetic architecture systematically explored [9]. The chicken genome is relatively compact, with an approximate size of 1.2 billion base pairs distributed across 39 pairs of chromosomes, including all 10 autosomal macrochromosomes, both the *Z* and *W* sex chromosomes, and two-thirds of the 28 microchromosomes [10]. In 2004, the chicken genome became the first avian genome to be sequenced. The sequencing was accompanied by the development of essential resources, such as Bacterial Artificial Chromosome (BAC) libraries and a BAC-based physical map [11], which allowed researchers to organize and analyze the genome systematically. The identification of 2.8 million single nucleotide polymorphisms (SNPs) further enhanced the genetic setting, enabling studies on genetic diversity and its association with traits of interest [12]. Despite its size being smaller than most mammalian genomes, the chicken genome contains a lot of information that has illuminated key aspects of vertebrate biology. The chicken genome in most cases has a 1:1 correspondence between homologous genes in mammals and birds, which includes a high level of sequence conservation in intronic and non-coding regions that are likely to contain important regulatory elements, allowing for extensive genetic analysis and comparison with humans, thus enabling the expansion of transgenic techniques in the CE model [1]. The main advantages of using CEs for experimental embryology are the ease of transplanting cell lines and tissues and their similarity to mammalian systems [13].

## 3. Development Stages

The development of CEs is a well-defined process that occurs in several stages [14]. Here is a developmental timeline with a comprehensive view of how a CE progresses from fertilization to hatching, highlighting key milestones in embryonic development.

1. Fertilization occurs in the oviduct of the hen after mating. The sperm fertilizes the egg, resulting in a zygote. The fertilized egg then begins its journey through the oviduct, where it accumulates layers of albumen (egg white), membranes, and the shell.
2. Cleavage and early development: the fertilized egg transforms into a blastoderm approximately 0–3 h after fertilization. The fertilized egg undergoes a series of rapid mitotic divisions, known as cleavage, resulting in a cluster of cells. These cells form a small disc on the surface of the yolk called the blastoderm or the blastodisc.
3. Gastrulation: the formation of germ layers occurs approximately 6–12 h after fertilization. The blastoderm undergoes gastrulation, forming three primary germ layers: the ectoderm (develops into the skin and the nervous system), the mesoderm (forms muscles, bones, and the circulatory system), and the endoderm (becomes the digestive tract and the internal organs).
4. Neurulation: the formation of the neural tube occurs around 24–48 h after fertilization. The ectoderm forms the neural plate, which folds to create the neural tube. This tube will eventually develop into the central nervous system (the brain and the spinal cord).
5. Organogenesis: the development of organs and tissues occurs from about 48 h to several days. The germ layers differentiate into various organs and tissues.
6. Morphogenesis: this occurs over 3–5 days until hatching. The embryo’s body undergoes changes to take on the recognizable form of a chick. Features, such as feathers, the beak, and talons, start to develop. The embryo’s organs become more defined and functional.
7. Hatching: preparation for birth occurs approximately 20–21 days after fertilization. The embryo becomes fully developed and ready for hatching. It absorbs the remaining yolk sac, which provides the nutrients needed to survive outside of the egg. The chick uses an egg tooth (a small, temporary structure on its beak) to break through the shell.

It is also possible to differentiate the stages according to phenotypic changes. In fact, in the early stages, the embryo is transparent and simple structures are visible, whereas in later stages more complex structures, like organs and limbs, become visible [15]. All of the steps are well-differentiated based on the time and days of development. We summarized all of the changes in the following table.

Developmental stages can also be observed in vitro when CE is used as an experimental model. To this aim, the fertilized egg must be placed in a specialized incubator. This incubation system is designed to automatically regulate the temperature and humidity. The most critical component of incubation is maintaining a constant temperature of 37.7 °C, which is essential for embryo development over a specified period. Additionally, maintaining a consistent humidity level of 47% is crucial; if the surrounding air is too dry, the egg can lose excessive water, thus hindering or preventing development [16].

The artificial incubation process offers several advantages:

- It allows for planned development and hatching;
- It prevents the spread of diseases and parasites;
- The likelihood of egg spoilage is minimized because all eggs are kept at optimal hatching temperatures;
- It eliminates the risk of hens damaging the eggs by pecking, which is common in natural incubation [17].

**Figure 1:**
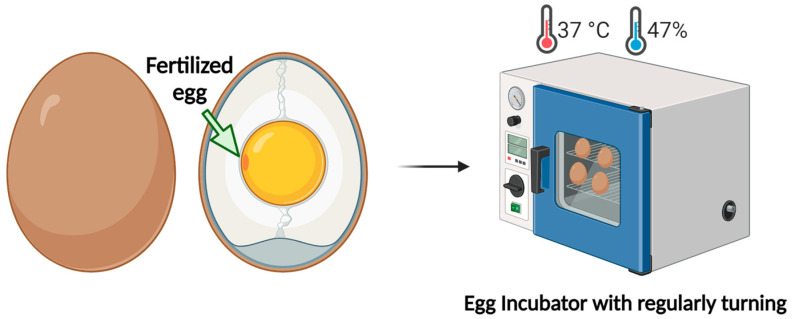
Incubation system for CE as experimental model. (Created with BioRender.com).

It has been reported that development begins the moment the fertilized eggs are placed in the incubator, that is, day 0 [18]. From this point on, development begins with the introduction of the eggs into the incubator. From this time, the blastoderm is formed, and gastrulation begins (Table 1). During incubation, it is essential to turn the eggs regularly. Turning prevents the embryo from floating and adhering to the shell, which would complicate further work with the embryo. This practice also prevents premature adhesion of embryo membranes, helps the embryo move into the correct developmental position (thereby reducing abnormalities and malposition), stimulates membrane growth, and increases the heart rate. The increased heart rate and membrane growth enhance nutrient uptake and improve the exchange of oxygen and carbon dioxide within the egg [18] (Figure 1).

### The Hamburger–Hamilton (HH) Stages

The Hamburger–Hamilton (HH) stages are a widely accepted system for describing the development of CEs. Developed by Viktor Hamburger and Howard L. Hamilton in 1951, this staging system provides a detailed and standardized framework to describe embryonic development from the earliest stages to hatching [4]. The HH staging system divides chicken embryonic development into 40 stages, each representing specific developmental milestones and features (Table S1).

## 4. Chorioallantoic Membrane (CAM)

**Figure 2:**
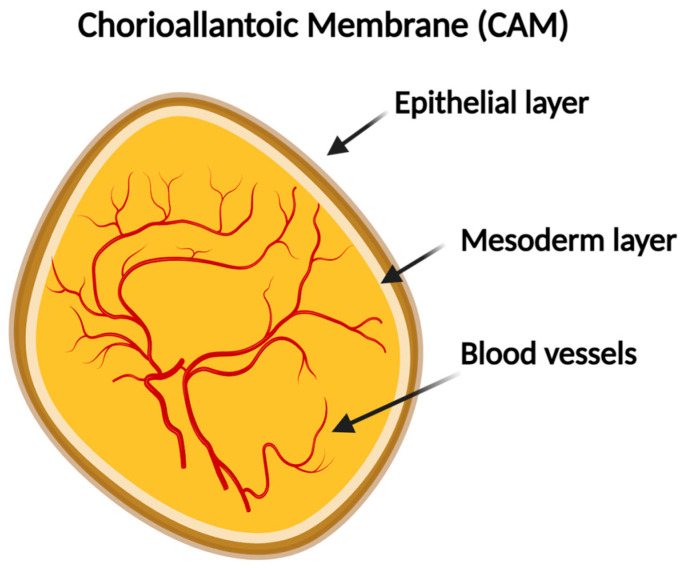
Graphical illustration of the CAM. The CAM comprises two different layers and a blood vessel system (Created with BioRender.com).

The CAM of the CE is a vital structure that supports embryonic development by enabling efficient gas exchange, nutrient absorption, and waste elimination [19]. It is formed through the fusion of two membranes: the chorion, which is the outermost layer that develops from the trophoblast and initially forms around the embryo, and the allantois, which is an outgrowth of the embryonic posterior intestine that extends into the extraembryonic coeloma and finally fuses with the chorion to form the CAM. On the other hand, the mature stage of the CAM comprises three distinct layers. At the surface, there is an epithelial layer composed of epithelial cells. Below that, the mesoderm layer contains blood vessels and connective tissue, and deep down, there is a dense network of blood vessels that supplies oxygen and nutrients to the developing embryo and is critical for the study of angiogenesis (Figure 2) [19].

Due to its high vascularity, the CAM facilitates the exchange of oxygen and carbon dioxide between the embryo and the external environment through the eggshell. It is also involved in the transfer of calcium from the eggshell to the developing embryo, which is essential for the formation of bones and other structures, and in the removal of waste due to the allantoic portion acting as a reservoir for waste products, particularly uric acid, produced by the embryo. This helps to keep the embryonic environment clean and suitable for development [20]. The developmental timeline of the CAM begins in the early stages of incubation. Around HH Stage 8, which corresponds to approximately 72–84 h of incubation, the allantois starts to form. By HH Stage 15, approximately 156–168 h into incubation, the allantois has extended and fused with the chorion, resulting in the formation of the CAM. During the late stages of development, the CAM continues to expand and becomes fully functional, playing a crucial role in gas exchange and nutrient transfer until the chick is ready to hatch. Experimental techniques involving the CAM begin with setting up the CAM assay. To expose the CAM without disturbing the embryo, a small window is created in the eggshell, allowing for direct observation and manipulation. Tumor cells, drugs, or other substances can be grafted onto or into the CAM to study its effects.

For observation and analysis, various microscopy techniques, such as bright field, fluorescence, and confocal microscopy, are used to visualize blood vessels and other structures within the CAM [21]. In addition, vessel density, branching, and other parameters are measured to evaluate the effects of experimental treatments. The CAM is used in a variety of research applications. In angiogenesis studies, it serves as a valuable model to observe and quantify the formation of new blood vessels in response to various stimuli or treatments. It is also used in drug testing to assess the impact of anti-angiogenic drugs or other compounds on blood vessel growth. In cancer research, implanting tumor cells on the CAM allows for studying tumor growth and vascularization, which is critical for understanding tumor progression and spread [22]. In developmental biology, the CAM is used to explore vascular development and the formation and regulation of blood vessels during embryonic growth. It also provides information on the effects of genetic changes on vascular development and embryogenesis [23].

In this scenario, the relevance of the CAM to human health is significant because the mechanisms of angiogenesis observed in the CAM are like those in human tissues [24]. This similarity allows researchers to explore the fundamental processes of blood vessel formation and function, which are relevant to various human conditions, including cancer and cardiovascular disease. Ethically, the CAM model presents fewer problems than vertebrate or higher mammalian models. The rapid development of the CAM also allows experiments of relatively short duration to be conducted, reducing the time and potential inconvenience associated with prolonged studies in more complex animal models.

## 5. Legislative and Ethical Issues

Within the framework of the 3Rs principles, the use of CEs in biomedical research is an excellent example of partial replacement [25]. Until a specific stage of their embryonic life, CEs are not deemed capable of experiencing pain [26]. Indeed, the European Directive 2010/63/EU exempts the use of avian embryos at the earliest stages of development from its scope [27]. Specifically, avian embryos are not subject to the same regulations as live animals until they are capable of independent life and experiencing pain, which typically corresponds to the last third of the incubation period [26,28,29]. However, determining the exact developmental stage at which embryos start pain perception represents a complex subject that intersects with developmental biology, neuroscience, and ethics [30]. Although the nervous system begins to develop at the end of embryonic day 1 (ED1), the anatomical structures involved in pain perception are not fully formed before ED13, coinciding with the development of a functional brain [4,31,32,33].

However, it is not guaranteed that the CE perceives pain only due to the presence of these structures, because the associated functional pathways must also be considered [34]. Electroencephalogram (EEG) onset has been identified at ED13, and recent studies have monitored pain responses in CEs at various developmental stages [28,29]. When exposed to noxious stimuli, alterations in arterial pressure and heart rate, as well as behavioral responses (such as movements of the beak and eyes), began around ED16 [29,35]. These physiological features must be considered during the design of experimental protocols involving CEs. Anesthesia should be administered during procedures for embryos at developmental stages above ED13 [36,37]. Anesthetic protocols for younger embryos are reported to induce immobilization and avoid motion artifacts during imaging studies [38,39].

Different euthanasia methods are recommended based on the developmental stage of the embryo. While hypothermia represents a suitable method for embryos before ED13, embryos from ED14 onwards could already be capable of experiencing suffering and should consequently be euthanized with humane methods [36]. Therefore, the use of CEs in biomedical research offers a valuable alternative to more sentient animal models by exploiting the developmental stages of embryos before pain perception is possible.

## 6. Manipulation of the CEs In Ovo and Ex Ovo

**Figure 3:**
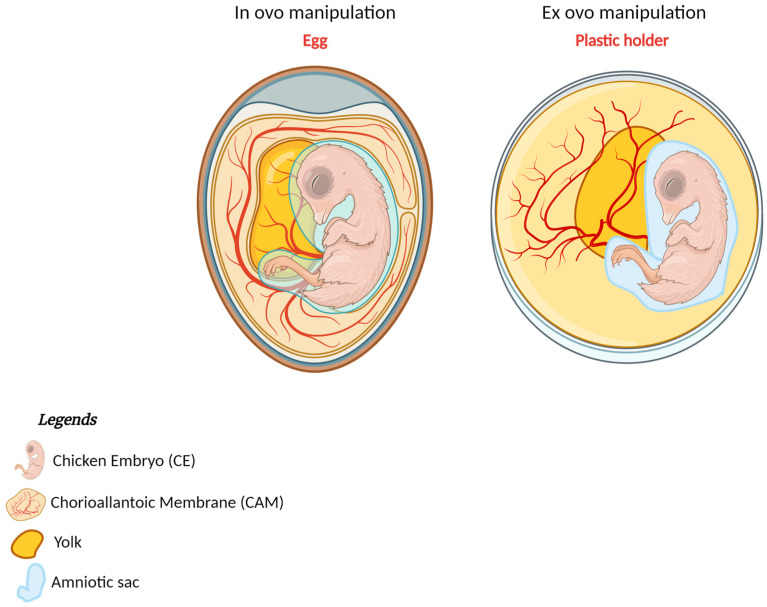
Manipulation of the CE. In ovo: development of the CE within the eggshell; ex ovo: CE growing outside of the natural eggshell, e.g., in a Petri dish (as in the figure) or a plastic holder. (Created with Biorender.com).

In studies involving CEs, both “in ovo” and “ex ovo” techniques are employed. The in ovo technique involves manipulating the embryo within the egg, while the ex ovo technique entails extracting and culturing the embryo outside of the egg [40]. The primary method for in ovo handling of CEs includes incubating and eggs’ fenestration. During incubation, fertilized chicken eggs are maintained at 37.7 °C with 47% humidity. Fenestration, which consists of creating a small window in the eggshell to access the embryo, is usually performed with a dental drill or small scissors. For ex ovo manipulations, the embryo is removed from the egg and cultured externally in new culture environments, including an eggshell surrogate, Petri dishes, and artificial eggshell-like vessels (Figure 3).

In ovo manipulation maintains the embryo within its natural egg environment, providing a more realistic developmental context but with limited accessibility. Ex ovo manipulation, on the other hand, allows for greater experimental control and accessibility but involves culturing the embryo in an artificial environment. Each method has its own advantages and limitations, making them suitable for different types of studies in developmental biology (Table S2). The ex ovo method involves explanting and culturing the CE in vitro by immersing the egg contents in a salt solution, separating the embryo from the yolk while preserving as much of the vitelline membrane as possible, and then transferring the embryo into culture until it reaches HH18. Due to the high skill level required, the lengthy process, and the difficulty of adapting the method for time-lapse imaging, Chapman et al. and Rupp et al. modified Denis New’s technique to simplify and speed up the process [41,42]. Their modifications addressed the complexities of the original technique, reduced the required skill level, eliminated the need for glass rings, and made the method easily adaptable for microscope imaging and other applications [43]. These advances have made possible the manipulation and observation of CEs for various research purposes, improving the understanding of embryological development and facilitating the advancement of medical research. Ex ovo techniques are extensively used in developmental biology to study the mechanisms of embryogenesis, organ development, and differentiation.

It is possible to observe the direct effects of genetic and environmental manipulations on developing embryos. The in ovo model is primarily used to study tumor cell extravasation and metastasis, while the ex ovo model is more suited to angiogenesis studies due to easier observation of the CAM [44].

Several in ovo and ex ovo techniques are used, including microinjection, electroporation, surgical manipulations, and molecular imaging.

*Microinjection* involves introducing substances, such as DNA, RNA, proteins, and dyes, directly into the embryo or yolk.

*Electroporation* uses electric fields to introduce DNA or other molecules into cells to study gene function in CEs, and it has demonstrated valuable application in developmental biology and disease modeling.

*Molecular imaging*, combined with in ovo and ex ovo techniques, allows for real-time visualization and manipulation of biological processes within living organisms. The ex ovo method is compatible with time-lapse imaging using light or confocal microscopy, as the embryo develops in an optically clear environment that does not scatter light. However, it has the disadvantage of short and low embryo survival and a high mortality rate of approximately 50%. In contrast, the in ovo technique offers better survival rates (65–80% depending on the stage of development) and allows for longer culture periods, although it is less suitable for in vivo imaging [43].

## 7. The Use of CEs in Preclinical Research

The first study of CEs dates back to 1911 when Rous et al. and Murphy et al. demonstrated how it was possible to transplant the chicken sarcoma onto the CAM and observe its growth [45]. In the following years, several researchers demonstrated the potential use of CEs as an animal model for implantation of tumor cells and tissues [46,47,48]. Nowadays, the CAM model not only provides a unique three-dimensional environment to observe and study tumor growth and metastatic invasion in real time, but it can also be used to evaluate angiogenesis, as different vascular patterns can be observed during embryonic development, allowing researchers to test the efficacy of new drugs and compounds.

**Figure 4:**
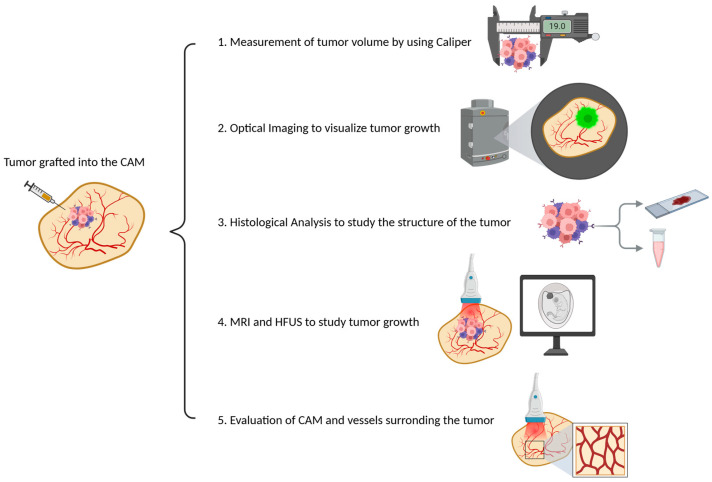
Several methods to assess tumor growth on the CAM. (Created with Biorender.com).

Tumor mass in CE models can be measured using the following methods:

- Direct measurement of tumor volume using calipers;
- Optical imaging to visualize tumor growth in real time;
- Histological analysis to study the structure of the tumor at the cellular level;
- Magnetic resonance imaging (MRI) and high-frequency ultrasound (HFUS) to monitor tumor growth and detailed anatomical information (Figure 4).

In addition, the angiogenesis in CAM models can be quantitatively and qualitatively assessed using the following methods:

- Microvascular density analysis that allows researchers to visualize blood vessels and calculate the density of microvessels in both the tumor and the surrounding tissue;
- Fluorescent labeling and imaging through fluorescent labeling of tumor cells and blood vessels that allow for real-time tracking of vessel growth using confocal microscopy, providing insight into how tumor cells modify their microenvironment to promote angiogenesis;
- Repetitive color-duplex-ultrasonography, which was used for the analysis of tumor growth and tumor vascularization, allowing for the quantification of tumor size and the monitoring of angiogenesis [49] (Figure 4).

**Figure 5:**
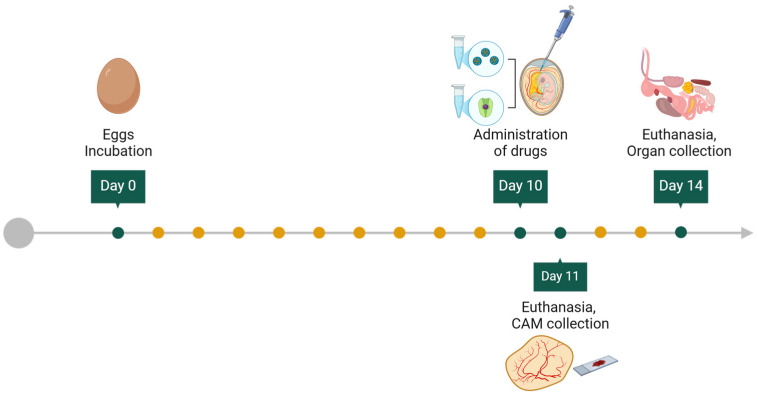
Drug delivery system setup. Schematic representation of drug conjugates’ administration using carriers or nanoparticles. (Created with Biorender.com).

CEs could be used as a drug delivery system (DDS) to deliver therapeutic agents to specific sites of injury or disease. The transition from cultured cells in vitro to preclinical animal models in vivo is complicated and has a low success rate compared with expected results. In this scenario, the CAM has proven to be an excellent alternative to test a DDS in a model considered intermediate between in vitro analysis and preclinical in vivo validation in animal models [50]. The CAM model can be used to test pro-angiogenic drugs in the context of tissue repair after an ischemic insult by controlling angiogenesis directed at the damaged site. These drugs, which are mainly pro-angiogenic factors or their analogue, can be administered in different modalities as drug conjugates using various carriers, such as micro or nanoparticles. A major advantage is the possibility of the administering candidate compound directly into the membrane with a localized application that minimizes systemic effects in the embryo [23,51,52,53] (Figure 5). The importance of the CAM assay for angiogenic drug development lies in its translational value; the molecular pathways that drive angiogenesis in CEs, such as Vascular Endothelial Growth Factor (VEGF) and Fibroblast Growth Factor (FGF), are the same as those underlying human neo-angiogenesis. Drugs with anti-angiogenic action can be studied using the CAM model as the optimal platform for direct observation of the response in terms of inhibition of neo-vascularization or with regard to their efficacy in inhibiting tumor neo-angiogenesis, as previously described [54].

In the context of angiogenesis studies, for example, Ribatti et al. described the use of the CE CAM model as an in vivo platform for studying human neuroblastoma [55]. The grafted tumor cells interact with the dense network of blood vessels and stimulate the formation of new vessels infiltrating the tumor, a process identical to what occurs in human tumors. The authors after 7 days of incubation excised the tumor surrounding the CAM tissue and fixed it for histological analysis to assess tumor-induced angiogenesis [55].

Lokman et al. demonstrated the application of the CAM model for studying ovarian cancer growth, angiogenesis, and drug testing, highlighting its utility for screening new anti-cancer therapies [56]. The authors measured the tumor size with a caliper or imaging software over time, while angiogenesis was assessed by visually examining and quantifying the number of new blood vessels forming around the tumor. The authors used anti-angiogenic therapeutic agents directly on the CAM and evaluated their effects on ovarian tumor growth and angiogenesis. Treated tumors showed a significant reduction in size compared with untreated controls, reduced cell proliferation within the tumor, as evidenced by decreased Ki-67 staining, along with a reduced number of blood vessels forming around the tumor, as demonstrated by decreased microvascular density (MVD) in histologic analysis. Dagg et al. demonstrated that the human epidermoid carcinoma cell line implanted on the CAM spontaneously metastasizes to various organs, including the eye, brain, liver, and myocardium [57], and that chicks hatched from eggs inoculated with tumor cells died within a few weeks due to the development of tumors in the brain, liver, heart, and kidney. Easty et al. made it possible to detect and quantify micro-metastases by measuring the distribution of cells from several 3H-labeled murine tumor lines [58]. Endo et al. standardized a sensitive method for the specific detection of tumor cells present at secondary sites using Polymerase Chain Reaction (PCR) with primers specific for the β-globin gene [59]. Next, the migration of tumor cells in different organs of the CE was studied using intravital imaging [60,61]. Deryugina et al. [60,62,63], used fluoresceinated HT-1080 human fibrosarcoma cell variants along with vasculature labeling with Lens Culinaris Agglutinin (LCA) to follow with real-time imaging the intravasation, dissemination, and extravasation of tumor cells with different dissemination potentials. Ronca et al. used histological methods to visualize mock cells and previously transduced human pentraxin 3 (hPTX3-B16-F10) cells and quantify their invasive capacity through the CAM to the underlying mesoderm. For this purpose, melanoma cells possibly present in the CAM mesenchyme were counted 4 days after grafting, and the results showed a significantly reduced number of hPTX3-overexpressing cells compared with mock cells [64]. These data confirmed using the CAM model that PTX3 inhibits Epithelial–Mesenchymal Transition (EMT) in melanoma cells, reducing their metastatic potential. Lugassi et al. used the CAM to explore the ability of angiotropic melanoma cells to metastasize by migrating to secondary sites [65]. In particular, the authors described the implantation of C8161 human melanoma cells labeled with Green Fluorescent Protein (GFP) on the CAM and their invasion into distant organs of the CE by using fluorescence microscopy. In parallel experiments, they performed PCR analysis to detect human cancer cells in the organs of chicks.

In the field of DDSs, Guedes et al. used a breast CAM model obtained by engrafting MDA-MB-231 cells to test the effect of different anti-tumor heterobimetallic Ru(II)/Fe(II) complexes including two anti-angiogenic ones. They demonstrated that the CAM model is an optimal platform for evaluating their antiangiogenic complexes because of the ease with which the vasculature density can be observed to thus quantify the inhibitory effect in terms of its decrease in response to administration [66].

## 8. In Ovo and Ex Ovo Applications in Molecular Preclinical Imaging

Non-invasive preclinical imaging represents an essential tool to develop modern and translational biomedical research. Recently, imaging studies have focused on mice and rats due to their analogy to human systems, which can also be applied to other animal models, such as CEs, which show genetic similarity with humans up to 80%, compared to 75% between humans and zebrafish and 60% between humans and melanogaster [67]. In detail, imaging of CEs and the CAM has emerged as a preclinical in vivo model that bridges the gap between in vitro and in vivo studies [68]. Consequently, it is not a replacement for the mouse model, but it could be considered as a preliminary step to in vivo studies using more neurologically developed animals compared to cells, with lower management costs compared to mouse models. This provides the best conditions for translating the in vivo studies performed and reducing the number of animals to be used, according to the 3Rs principle. The combination of imaging with the study of CEs is characterized by several advantages. This model is cheaper than other commonly used animal species, there are no housing costs, and the space required to house the eggs consists of small incubators. Furthermore, physical access to the egg is easy because it has no barriers beyond the shell itself, allowing for small operations, such as the implantation of tumor cells and the injection of fluorescent probes/microbubbles/contrast agents [69].

In ovo and ex ovo techniques are used in combination with advanced molecular imaging both to gain information about the development of the CE by studying embryonic development, gene function, and molecular interactions and to study human diseases and treatment responses. In the following sections, some examples where the various imaging methods are used for in ovo and ex ovo studies of CEs are shown.

### 8.1. Preclinical HFUS in CE Studies

**Figure 6:**
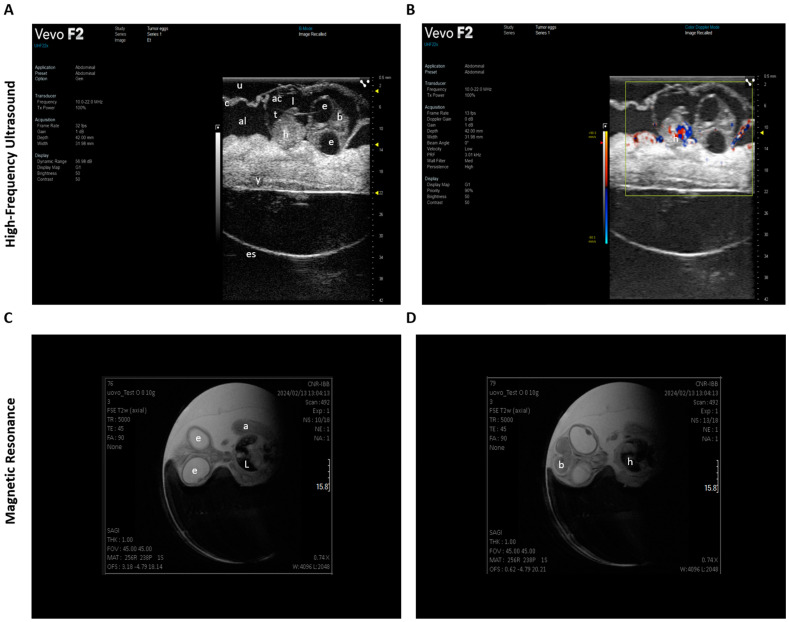
Multimodal imaging of CE. (**A**) B-mode and (**B**) color doppler mode transversal section at ED7, imaged with UHF 10–22 MHz transducer (acquisition parameters: frame rate 32 fps, depth 42.00 mm, width 31.98 mm, transmitted power 100%, dynamic range 56.98 dB) [Visual sonics Vevo F2 Ultrasound scanner]. (**C**,**D**) T2-weighted sequence to assess the organogenesis at ED13; the high soft tissue contrast of the MRI allows for identifying the different organs during the development of the CE [7T MR Solutions]. e: eye, a: abdomen, L: liver, b: brain, h: heart, t: thorax, l: limb, c: CAM, y: yolk, al: albumen, ac: amniotic cavity, u: ultrasound coupling gel, es: egg shell.

HFUS is a method widely used in the preclinical field. Indeed, most research in the oncology, metabolic, and embryological development fields has been developed using small animal models thanks to the use of HFUS. In particular, HFUS is a diffused method to study tumor biology, novel anti-cancer treatments, and angiogenesis in CE, so the peak frequencies exceeding 40 MHz of this imaging technique enable a higher resolution of micro vessels, which is essential for the in ovo visualization of CAM vasculature [70] (Figure 6).

Furthermore, in the cardiovascular field, the heart of the CE is the first organ that starts to work. Indeed, it beats at 33 h post-incubation, and the time of heart development is longer than in other species, allowing researchers to perform deeper longitudinal studies [71]. Adapting preclinical US systems and parameters, Hegemann et al. were the first to characterize the CE from ED 8 to ED 13 through B-mode and M-mode imaging, obtaining a four-chamber view as in clinical and pre-clinical practice. This study demonstrated that HFUSs on CEs can be a useful tool for evaluating the effect of drugs and innovative therapies on cardiopulmonary development while avoiding the sacrifice of further animal species with advanced neurological development [67].

In order to observe angiogenesis development during cystic tissue growth, Schueler et al. established human renal cystic tissue onto the CAM between the 7th and 10th days of embryonic development. HFUS allows for revealing the engraftment, increase of vascularization, and changes in lesion dimensions in real time and in non-invasive ways [70]. In vivo models of cystic tissue, in combination with imaging of anastomosis and angiogenesis development using HFUS, offers promising instruments to study personalized therapy [70].

The microvascular study of tumors in a preclinical setting requires high sensitivity in the detection of small vessel dimensions and blood flow. For this purpose, ultrafast Ultrasound Microvessel Imaging (UMI) was developed, which is essentially capable of increasing the doppler sensitivity at low flow speeds coming from micro vessels characteristic of the CAM. In the CAM model of a renal cell tumor, the HFUS system Vevo 3100 supporting UMI software was able to distinguish between the untreated tumor and the sunitinib-treated tumors, which were characterized by a more heterogeneous blood supply distribution, with a perfusion gradient starting from the center of the upper tumor surface (where the drug was administrated) to the periphery of the tumor. The main advantage of UMI is the ability to analyze the angiogenesis and vascularization in a tumor engrafted into the CAM of CEs while avoiding the technical difficulty of injection of microbubble contrast agents [72].

### 8.2. Preclinical MRI in CE Studies

In the last years, MRI, with the diffusion of high field scanners, has also gained importance in the field of preclinical research. MRI provides detailed morphological images conjugating excellent soft tissue contrast with high spatial resolution (up to 50 μm in animal studies) and temporal resolution. It also allows for the collection of information about tissue composition, perfusion, oxygenation levels, tissue elasticity, and metabolism, and, recently, it further evolved toward imaging of molecular events, which represents the true challenge for imaging techniques [73,74]. Thanks to its flexibility in contrasts, MRI already raised interest in imaging CEs in the 1980s [75] (Figure 6).

Several authors in the past years have focused their activity on the development of specific procedures and tools to optimize MRI of the CE, providing useful indications for successive studies. For example, to obtain good quality images, motion-induced image artifacts should be avoided, and this is usually obtained by placing the egg in ice chips between 10 and 90 min. Waschkies et al. [39] further explored the immobilization of the CE, comparing three anesthetic regimes consisting of dropping onto the surface of the CAM medetomidine at a dosage of 0.3 mg/kg, thiopental at 100 mg/kg, and ketamine/midazolam at 50 mg/kg and 1 mg/kg. The study resulted in better performances for medetomidine in terms of reduced motion, time of onset of anesthesia, and anesthesia duration. Another technical aspect to consider when performing CE MRI is the choice of the coil to optimize SNR and B1 homogeneity. Standard rat body birdcage coil is commonly employed, but it is not fully tailored to CEs in terms of size and length, and because the position of the CE changes over time, the organ of interest may not always be placed within the sensitive region of the coil. To avoid this issue, Choi et al. [76] introduced a new curved approach to coil design specifically tailored for CE MR scans, showing practical potential for in ovo studies. Holmes et al. [77] investigated the feasibility of a self-gated cine MRI protocol that incorporates a navigator-based retrospective gating technique to study CE hearts, overcoming the difficulty of monitoring chick ECG and respiration signals.

As for the methodological applications, thanks to its ability to differentiate soft tissues, MRI was used to monitor the development during embryogenesis of specific organs of interest. Zhou et al. [78] explored brain development using T2-Weighted Imaging (T2WI) for volume estimation and Diffusion Tensor Imaging (DTI) to reflect the evolution of neural bundle structures. Lindner et al. [79] studied chick eyes through T2WI, concluding that MRI has the ability to depict embryonic ocular development in a noninvasive and truly longitudinal manner. Finally, Chen et al. [80] evaluated the morphologic evolution of the CE, the allantois, and the CAM throughout ED1 until ED20, using T2WI and T1WI and semi-automated segmentation algorithm for volume computation.

In the field of human disease studies, thanks to the refinement of CE xenograft models, several applications using MRI have been described in oncology, for the development of nanoparticle-based carriers and imaging agents, for treatment evaluation, and for the optimization of imaging techniques. After the optimization of an acquisition protocol for tumor visualization composed by T2WI, DWI and T2 mapping sequences [81], Zuo et al. used MRI to compare the biodistribution of Gd-DOTA conjugated micelles to standard Gd-DOTA in xenografts of human breast cancer cells on the CAM, showing an improved signal-to-noise ratio with the micelles [82]. Hafner et al. [83] used MRI to investigate the localization of a nanocarrier composed of albumin and PEG loaded with doxorubicin and labeled with DOTA-Gd in triple-negative breast cancer, showing that the particles, approximately 100 nm in size, preferentially accumulated in xenograft tumors over time. Another intriguing application of MRI is the tracking of cells labeled with MRI-visible nano- and microparticles over time. SPION-labeled melanoma cells were injected into the neural tube of a CE, and their migration along the neural crest pathways was monitored for 16 days post-injection [84]. This method was also used for mesenchymal stem cells labeled with iron oxide nanoparticles implanted in the avian embryo’s brain. Notably, these mesenchymal stem cells were also tagged with a fluorescent protein enabling the co-registration of live cell locations via optical imaging, confirming particle retention within the cells [85]. Herman et al. [86] evaluated the advantages and limitations of MRI to study metastatic dissemination of neuroblastoma in the chick starting from MPIO-labeled neuroblastoma cells implantation. Buschmann et al. and Waschkies et al. tried to characterize different tumors in living CEs in terms of their reaction to gas challenges. They used T1 and T2* mapping to enable differential characterization of tumor grafts with respect to their vascular and oxygenation status [87,88].

An interesting utilization of MRI was developed by Kivrak Pfiffner et al. [89]. The authors used the CAM as an environment for 3D biomaterial scaffold proliferation and presented a novel in vivo method for analyzing their perfusion capacity, thus enabling the evaluation of bioengineered material properties through this imaging modality.

With the development of simultaneous the Positron Emission Tomography (PET)/MRI scanner, the combination of the multi-contrast capabilities of MRI with the outstanding sensitivity of PET appears promising for in ovo imaging. A very recent study [90] used a simultaneous scanner to study a patient-derived xenograft model generated from the liver metastasis of a colorectal cancer with [^68^Ga]Ga-Pentixafor.

### 8.3. Other Imaging Applications in CE Studies

To assess the metabolic pattern of tumor cells grown on CE models, Smith et al. performed dynamic PET imaging in ovo, producing a high tumor-background signal for both ^18^F-2-fluoro-2-deoxy-D-glucose (^18^F-FDG) and (4S)-4-(3-^18^F-fluoropropyl)-L-glutamate (^18^F-FSPG) to assess glucose metabolism and uptake. It was possible to delineate radiotracer uptake in both the tumor and the CE [91]. Zlatopolskiy et al. reported an efficient method for the preparation and biological evaluation of fluoro-tryptophan (Trp) labeled at different positions with ^18^F. They analyzed the bone metabolism with ^18^F-fluoride and the Trp metabolic pathway with 7-^18^F-fluorotriptophan (7-[^18^F] FTrp) on the CAM membrane, where the uptake mechanisms and dehalogenation levels were comparable to those in mice. Therefore, 7-[^18^F] FTrp was identified as a very promising PET probe for imaging of Trp metabolism [92]. Warnock et al. combined the in ovo CAM glioblastoma model with in vivo PET/CT imaging, offering an innovative, cost-effective, and ethically beneficial study. In particular, they successfully demonstrated PET imaging of glucose metabolism and protein synthesis in CAM glioblastoma U87 cell lines. By capturing the PET tracer ^18^F-FDG over time in individual tumors, they obtained information about glucose metabolism, while through CT imaging they improved the accuracy of tumor volume measurements [93]. Optical methods, such as Optical Coherence Tomography (OCT) and doppler techniques, have been successfully applied to derive functional and physiological properties of the embryo. Li et al. used an ultrafast 1310 nm dual-camera Spectral-Domain Optical Coherence Tomography (SDOCT) system to characterize in parallel the dynamic radial deformation rate of the myocardial wall and the doppler velocity of the underlying blood within a beating CE in vivo. Through this system, they obtained simultaneous characterization of tissue motion and blood flow [94]. Li et al. also demonstrated the possibility of revealing complex myocardial activity in the live chick heart using OCT. They performed a measurement of in vivo deformation and the myocardial strain rate by analyzing the periodic variation of myocardial wall thickness calculated from real-time serial OCT images [95]. Other studies performed on the CE have allowed for the evaluation of useful bone regeneration for tissue engineering purposes using the CAM as a bioreactor to culture and study live human bone regeneration.

Microcomputed tomography (μCT) was used to quantify the extent and location of bone volume changes, followed by histological analysis to assess bone repair. This human–avian system described above provides a simple refinement model for animal research and a step toward an in vivo humanized model for tissue engineering [96]. New methods for nuclear imaging were also transferred for in ovo studies, especially for the initial testing of new labeling strategies [91].

## 9. Conclusions

**Figure 7:**
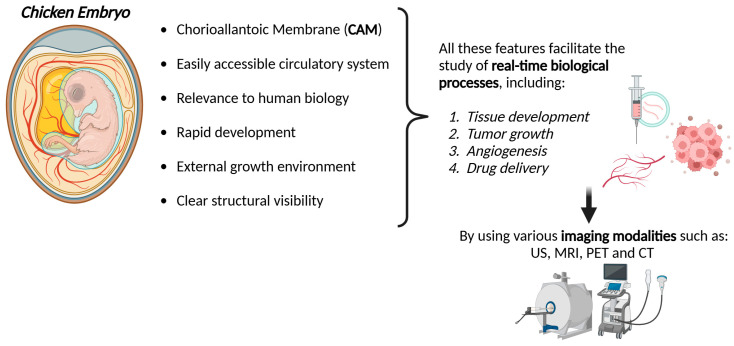
CE as a promising model for preclinical molecular imaging studies. (Created with biorender.com).

The CE model holds promise as a versatile and cost-effective alternative for preclinical molecular imaging and research, especially when compared with traditional mammalian models (Figure 7).

Its unique features, such as external development, ease of genetic manipulation, and ethical advantages, pose it as an ideal tool for studying developmental processes and real-time observation and imaging of complex biological processes, including tumor growth, angiogenesis, metastasis, and drug delivery. On the other hand, despite these advantages, the use of this model has several limiting disadvantages. The lack of a fully developed immune system in the CE model limits its ability to simulate the full complexity of human biology, particularly in terms of immune–tumor interactions. For early development stages studies, which do not require ethical approval, the time to conduct experimental procedures is very short, precluding long-term observations, which are essential for studying, e.g., tumor relapse and remission phenomena or response to therapies. Another challenge is the administration of molecules. Because oral administration is not an option, the CAM model requires the use of solubilized molecules to be injected, strongly limiting the types of compounds that can be tested using this model.

Despite these limitations and the difficulty of directly translating the obtained results to human biology, given the diversity between the two biological systems, the model’s detailed cellular dynamics, together with the possibility to monitor them through real-time imaging, provide insights that are useful in preclinical oncology, regenerative medicine, and neuroscience.

## Figures and Tables

**Table 1:** Overview of the developmental stages and key developments of CEs from day 1 to hatching.

Day	Developmental Stage	Key Developments
Day 1	Cleavage and early embryogenesis	Formation of the blastoderm and beginning of gastrulation
Day 2	Early organogenesis	Development of basic organ systems begins
Day 3	Heart formation and rudimentary organs	Formation of the heart and rudimentary organs; the embryo’s shape becomes more defined
Days 4–5	Continued organ development	Limb buds become more prominent; the embryo begins to resemble a chick
Days 6–10	Refinement of organ development	Further development of feathers and other distinguishing features
Days 11–14	Maturation	Further development of feathers, beak, and legs; internal organs mature
Days 15–18	Growth and preparation for hatching	The embryo grows and prepares to break through the shell
Days 19–21	Final preparation for hatching	The chick uses the remaining time to position itself and prepare for hatching

## Supplementary Materials

The following supporting information can be downloaded at https://www.mdpi.com/article/10.3390/biomedicines12122835/s1, Table S1: The Hamburger–Hamilton (HH) staging system in the chicken embryo (CE); Table S2: The main differences between in ovo and ex ovo manipulation.
