# Supplementary material for: The Chicken Embryo: An Old but Promising Model for In Vivo Preclinical Research
Source: Biomedicines. 2024 Dec 13;12(12):2835. doi: 10.3390/biomedicines12122835 (PMC11673736; doi:10.3390/biomedicines12122835)
Supplement: Supplementary file 1 [file biomedicines-12-02835-s001.zip › biomedicines-3337637-supplementary.pdf]

Supplementary Material

Table S1. The Hamburger-Hamilton (HH) staging system in Chicken Embryo (CE)

**Stages 1-10: Early Embryogenesis**
1. HH Stage 1 (Fertilization to 6 hours): the embryo is in the cleavage stage, with the fertilized egg starting to divide and form a blastoderm on top of the yolk
2. HH Stage 2 (Approximately 6-12 hours): the blastoderm forms and gastrulation begins, with the development of a primitive streak
3. HH Stage 3 (Approximately 12-24 hours): the primitive streak becomes more pronounced, the mesoderm begins to form, and the neural plate starts to appear
4. HH Stage 4 (Approximately 24-36 hours): the neural plate folds to form the neural tube, and somites, which will develop into vertebrae and muscles, become visible
5. HH Stage 5 (Approximately 36-48 hours): the neural tube closes, and head and tail folds are evident. Basic heart structures begin to form
6. HH Stage 6 (Approximately 48-60 hours): the body plan of the embryo becomes more recognizable, with limb buds forming and the heart starting to beat
7. HH Stage 7 (Approximately 60-72 hours): more defined head and tail regions appear, the eyes and ears begin to form, and limb development continues
8. HH Stage 8 (Approximately 72-84 hours): limb buds develop into more recognizable arms and legs, and the heart and circulatory system are further developed
9. HH Stage 9 (Approximately 84-96 hours): facial features become more distinct, limbs develop further, and digits begin to form
10. HH Stage 10 (Approximately 96-108 hours): the embryo continues developing facial features, limbs become more fully formed, and internal organ development progresses
**Stages 11-20: Organogenesis and Morphogenesis**
11. HH Stage 11 (Approximately 108-120 hours): organs become increasingly differentiated, the eyes are more developed, and limbs show distinct digits
12. HH Stage 12 (Approximately 120-132 hours): development of external features like feathers begins, and internal organs become more complex
13. HH Stage 13 (Approximately 132-144 hours): external features, including the beak and wings, become more prominent, clarifying the overall body plan
14. HH Stage 14 (Approximately 144-156 hours): feathers and other external features continue developing, and the heart and circulatory system are well developed
15. HH Stage 15 (Approximately 156-168 hours): features are well defined, the body becomes more recognizably chick-like, and feather development continues
16. HH Stage 16 (Approximately 168-180 hours): the body is almost fully formed, limbs are fully developed with distinct digits, and feathering is more pronounced

17. HH Stage 17 (Approximately 180-192 hours): the body and organ systems are nearly fully developed, and the external appearance is largely complete
18. HH Stage 18 (Approximately 192-204 hours): the body is mature, with well-developed feathers, beak, and limbs, and the chick prepares for hatching
19. HH Stage 19 (Approximately 204-216 hours): internal organs are fully functional, and the embryo begins positioning for hatching
20. HH Stage 20 (Approximately 216-228 hours): the chick is fully developed and ready to hatch, absorbing the yolk sac to provide nourishment immediately after hatching
**Stages 21-30: Final Maturation**
21. HH Stage 21 (Approximately 228-240 hours): the chick is ready to hatch, with fully mature internal processes and external structures
22. HH Stage 22 (Approximately 240-252 hours): final preparations for hatching are made, with the chick fully formed and positioned within the egg
23. HH Stage 23 (Approximately 252-264 hours): the chick begins the hatching process, breaking the eggshell and starting to emerge
24. HH Stage 24 (Approximately 264-276 hours): the hatching process is completed, and the chick emerges from the egg, fully formed and capable of independent movement
**Later Stages (31-40)**
These stages cover the period from just before hatching to post-hatching growth, during which the chick adapts to life outside the egg and gradually develops more complex behaviors and physiological functions.

Table S2. The main differences between *in ovo* and *ex ovo* manipulation

Aspect	*In ovo* manipulation	*Ex ovo* manipulation
**Environment**	Embryo remains inside the egg	Embryo is removed and cultured outside the egg
**Accessibility**	Limited by the eggshell; access through a small opening	High accessibility; direct access to the embryo
**Surgical Precision**	More challenging due to restricted access	Easier and more precise due to direct access
**Imaging and Observation**	Limited by the curvature and opacity of the eggshell	Enhanced imaging capabilities with direct visualization
**Application Complexity**	Often requires minimal disturbance to the egg	More complex setup for culturing the embryo externally
**Developmental Context**	Embryo develops in a more natural environment	Embryo develops in an artificial culture medium
**Survival and Viability**	Higher risk of infection and developmental disruption	Risk of culture-related artefacts and viability issues
**Experimental Control**	Less control over the external environment	Greater control over the environment and experimental conditions
